# Stable readout of observed actions from format-dependent activity of monkey’s anterior intraparietal neurons

**DOI:** 10.1073/pnas.2007018117

**Published:** 2020-06-24

**Authors:** Marco Lanzilotto, Monica Maranesi, Alessandro Livi, Carolina Giulia Ferroni, Guy A. Orban, Luca Bonini

**Affiliations:** ^a^Department of Psychology, University of Turin, 10124 Turin, Italy;; ^b^Department of Medicine and Surgery, University of Parma, 43125 Parma, Italy;; ^c^Department of Neuroscience, Washington University in St. Louis, St. Louis, MO 63110

**Keywords:** parietal cortex, action observation, visual invariance, neural decoding

## Abstract

Humans accurately identify observed actions despite large dynamic changes in their retinal images and a variety of visual presentation formats. A large network of brain regions in primates participates in the processing of others’ actions, with the anterior intraparietal area (AIP) playing a major role in routing information about observed manipulative actions (OMAs) to the other nodes of the network. This study investigated whether the AIP also contributes to invariant coding of OMAs across different visual formats. We recorded AIP neuronal activity from two macaques while they observed videos portraying seven manipulative actions (drag, drop, grasp, push, roll, rotate, squeeze) in four visual formats. Each format resulted from the combination of two actor’s body postures (standing, sitting) and two viewpoints (lateral, frontal). Out of 297 recorded units, 38% were OMA-selective in at least one format. Robust population code for viewpoint and actor’s body posture emerged shortly after stimulus presentation, followed by OMA selectivity. Although we found no fully invariant OMA-selective neuron, we discovered a population code that allowed us to classify action exemplars irrespective of the visual format. This code depends on a multiplicative mixing of signals about OMA identity and visual format, particularly evidenced by a set of units maintaining a relatively stable OMA selectivity across formats despite considerable rescaling of their firing rate depending on the visual specificities of each format. These findings suggest that the AIP integrates format-dependent information and the visual features of others’ actions, leading to a stable readout of observed manipulative action identity.

## Significance

The anterior intraparietal area (AIP) is a crucial hub in the observed manipulative action (OMA) network of primates. While macaques observe manipulative action videos, their AIP neuronal activity robustly encodes first the viewpoint from which the action is observed, then the actor’s body posture, and finally the observed-action identity. Despite the lack of fully invariant OMA-selective single neurons, OMA exemplars could be decoded accurately from the activity of a set of units that maintain stable OMA selectivity despite rescaling their firing rate across formats. We propose that by integrating signals multiplicatively about others’ action and their visual format, the AIP can provide a stable readout of OMA identity at the population level.

View-invariant neural processing of complex static stimuli, such as faces (1), objects (2), and body postures (3), can be achieved by different regions of primates’ inferotemporal cortex, despite a variety of visual presentation formats (4, 5). In the case of observed actions, the task is even more complex: In addition to changes in the visual format, such as viewpoint or actor’s body posture, others’ actions dynamically change their retinal images, making the extraction of a stable identity particularly challenging (6).

Humans can accurately discriminate observed actions irrespective of the viewpoint (7, 8). This ability seems to rely on a network of anatomically interconnected regions (9–11). Indeed, view-dependent neural encoding of others’ actions has been reported in the ventral (12–14) and mesial (15, 16) premotor cortex, prefrontal cortex (17), inferior parietal cortex (18), and, of course, superior temporal sulcus (19, 20), which represents the primary source of visual information for the parietofrontal action observation network (21) via the anterior intraparietal area (AIP).

Recent studies showed that the AIP is a core region of this network. In contrast to previous studies, which focused only on observed grasping actions, in a recent work we showed that the monkey AIP hosts neurons encoding specific observed manipulative actions (OMAs) and routes this information to the other nodes of the network (11). Furthermore, studies on the human homolog of the monkey AIP (22) have shown its specificity for the encoding of observed manipulative actions over other classes of actions, such as locomotion (23) or skin-displacing actions (24). Nonetheless, whether and how AIP neurons can also contribute to the representation of OMA identity across visual formats remain completely unknown.

To address this issue, we chronically recorded neuronal activity from the AIP of two macaques while they observed videos portraying seven manipulative action exemplars (drag, drop, grasp, push, roll, rotate, squeeze) in four distinct visual formats. The formats resulted from the combination of two actor’s body postures (standing, sitting) and two viewpoints (lateral, frontal). We found that AIP neuronal activity provides first a robust population code for viewpoints and actor’s body postures and then exhibits specificity for OMA exemplars. Despite the lack of fully invariant representation of OMA identity at the individual-unit level, we found that AIP neurons multiplicatively integrate signals about visual format and OMA identity. The neural population activity allowed us to decode action exemplars regardless of the visual format thanks to the activity of a set of units that maintain stable OMA selectivity but rescale their firing rate across formats. By integrating format-dependent visual information and the visual features of others’ actions, the AIP can provide a stable readout of OMA identity at the population level.

## Results

**Fig. 1.**
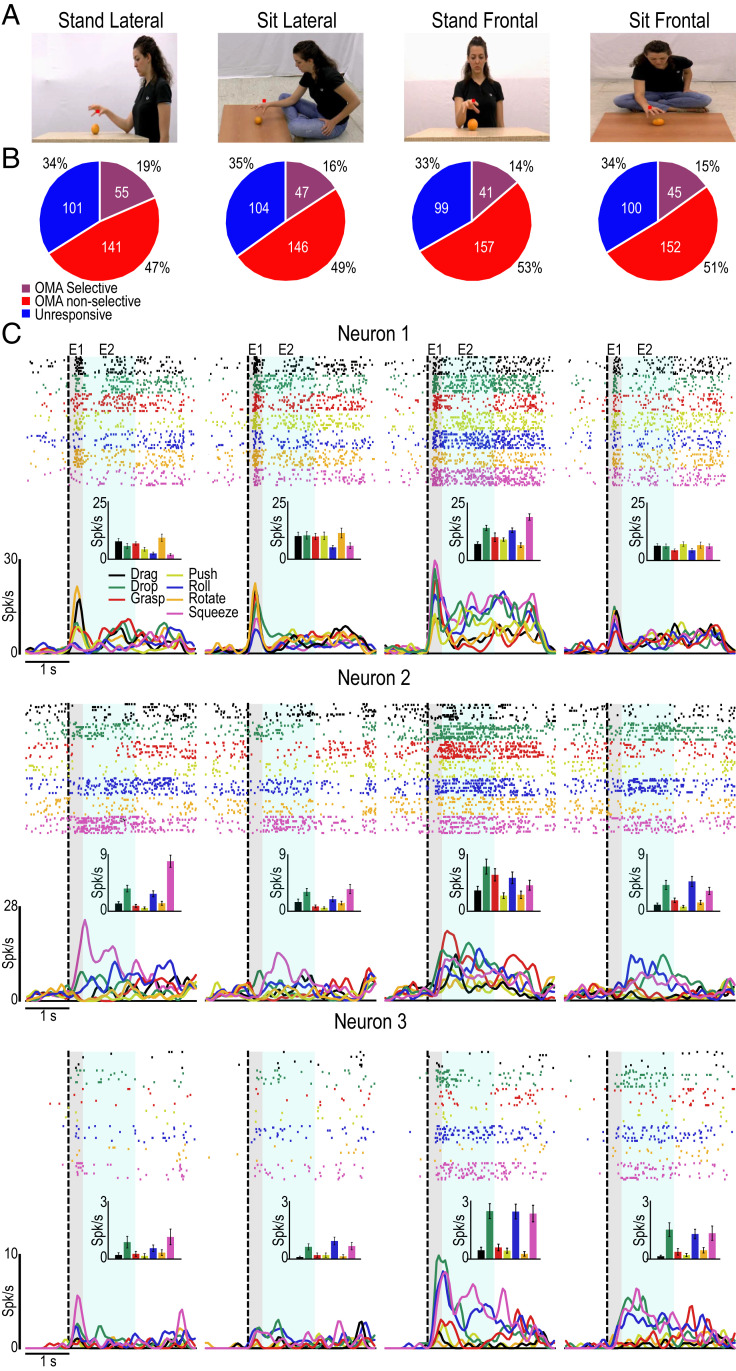
Tuning properties of AIP units and single-neuron examples while viewing OMAs in different formats. (*A*) Example frames taken from videos of grasping illustrating the four tested formats for OMA presentation. (*B*) Tuning properties of the recorded units in each of the formats. (*C*) Responses of three AIP OMA-selective single neurons to the seven OMAs (color-coded) in the four formats (example frames; *Top*). In addition to raster plots and peristimulus time histograms (PSTHs; 50-ms bins), the average firing rate (±SE) in epochs 1 (E1) and 2 (E2) is shown (*Insets*). Note that neurons 2 and 3 exhibit similar selectivity patterns in different formats, despite rescaling their firing rate in different formats.

We recorded AIP neuronal activity in two macaque monkeys (Mk1 and Mk2) from the same chronic implants described in a previous study (11). During the recordings, monkeys maintained fixation on the center of a screen while viewing videos depicting seven OMA exemplars (*Materials and Methods*) in four visual formats (Fig. 1*A*). The formats resulted from the combination of two postures (standing, sitting on the floor) and two viewpoints (lateral, frontal).

We isolated 297 units (72 single units and 225 multiunits), of which 182 were recorded in Mk1 (58 single units and 124 multiunits) and 115 in Mk2 (14 single units and 101 multiunits). Out of the 297 units, 64 were task-unrelated and 233 were action-related. Of these latter, 113 (38%) were OMA-selective in at least one format (*Materials and Methods*). As far as the four visual presentation formats are concerned, 50 OMA-selective units (44%) showed their selectivity in multiple formats, and the proportion of selective units was equally distributed among the formats (Fig. 1*B*; χ^2^, *P* > 0.05 for all possible combinations). By considering OMA selectivity within each format, we found temporally stable representation of OMA identity in each investigated format, separately (*SI Appendix*, Fig. S1), in line with our previous study (11) carried out with a single format (sitting at a table).

Notably, none of the recorded units showed OMA specificity completely invariant across all formats. Instead, single-neuron examples clearly demonstrate that the response pattern and tuning for OMAs varied considerably across formats. Some neurons (i.e., neuron 1, Fig. 1*C*) showed strong tuning for a specific format and OMA selectivity in that format, which is compatible with partially mixed selectivity (25), whereas others (i.e., neurons 2 and 3, Fig. 1*C*) exhibited a clearer and more repeatable preference for a few OMA exemplars in all formats, despite considerably rescaling their firing rate across formats.

### Visual Preference for OMAs in the AIP: Control Experiment.

**Fig. 2.**
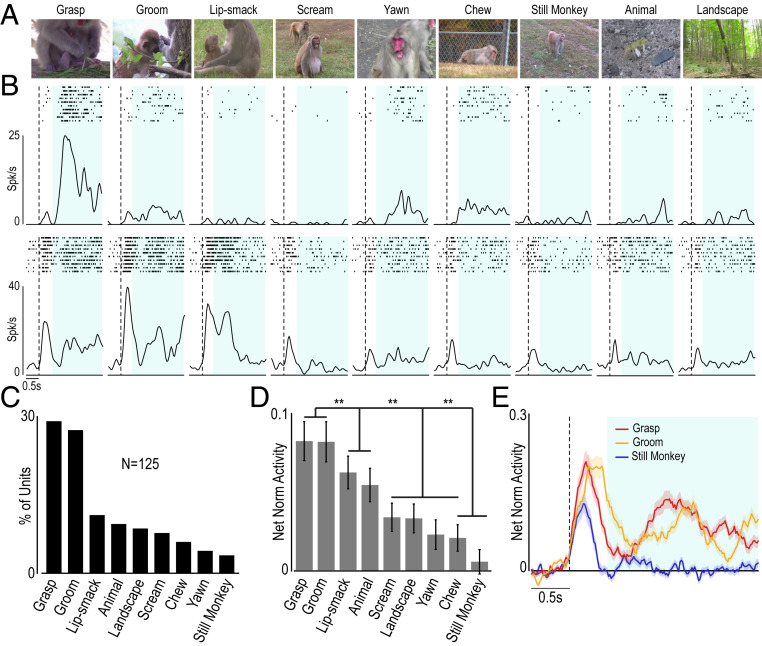
Control experiment. AIP preference for OMA relative to other natural video stimuli. (*A*) Frames taken from each of the nine presented videos. (*B*) Rasters and PSTHs illustrating the response of two AIP video-selective single neurons (in rows) to the nine video stimuli illustrated in *A*. Light blue shading indicates the epoch of interest for statistical analysis. Other conventions are as in Fig. 1*C*. (*C*) Frequency distribution of selective units based on their preferred video (χ^2^ = 67.62, *P* < 0.001). (*D*) Population response of all selective units to each of the presented videos. A one-way repeated-measures ANOVA followed by a Bonferroni post hoc test revealed no significant difference between grasp and groom, which in turn evoked a stronger discharge relative to all of the remaining stimuli. (*E*) Temporal profile of the selective units’ response to the preferred (grasp and groom) and not preferred (still monkey) videos. The shading around each curve corresponds to 1 SE. **P < 0.05.

The findings and single-neuron examples so far presented might suggest that AIP neuronal activity encodes low-level visual features, which vary across formats, rather than OMA identity. To address this issue, we carried out a control experiment in which we presented Mk1 with nine videos depicting natural dynamic scenes, varying considerably in low-level visual features, such as amount of motion and contrast (*SI Appendix*, Fig. S2 *A* and *B*). The set of videos included (Fig. 2*A*) monkey manipulative actions (grasp and groom), emotional facial gestures (lip smack and scream), neutral facial gestures (yawn and chew), a still monkey, a moving animal, and a dynamic landscape (*Materials and Methods*).

We recorded 215 units during random presentation of these videos in two sessions (104 in session 1 and 111 in session 2). Out of them, by comparing baseline activity with that during video unfolding (from 0.5 to 3 s following video onset; *SI Appendix*, Fig. S2*C*), we found 148 units specifically responding during video presentation (2 × 9 repeated-measures ANOVA; factor: epoch and video; Fisher least significant difference [LSD] test, *P* < 0.05). We excluded 23 units (15.5%) from further analyses because their firing rate was significantly correlated with the amount of motion characterizing different videos (*SI Appendix*, Fig. S2*D*). In contrast, none of the isolated units was significantly influenced by the amount of contrast in the videos (*SI Appendix*, Fig. S2*E*). Thus, the remaining 125 units exhibited specific tuning for the content of the videos (see example neurons in Fig. 2*B*), with a clear-cut prevalence of units (*n* = 66, 53%) with a stronger discharge during the observation of manipulative action (groom and grasp) among the nine tested videos (Fig. 2*C*). Consistently, population activity was significantly stronger for grasp and groom as compared with all of the other videos (Fig. 2 *D* and *E*). Importantly, low-level visual features cannot explain the greater preference of AIP units for both groom and grasp videos because they were considerably different from each other in terms of amount of motion (*SI Appendix*, Fig. S2*A*) and contrast (*SI Appendix*, Fig. S2*B*) and do not constitute outliers with respect to the other videos regarding both these parameters.

In sum, the control experiment and analyses presented above demonstrate that the AIP exhibits a clear preference for OMAs over other types of dynamic stimuli, and this preference cannot be accounted for by low-level visual features (see also *SI Appendix*, Fig. S1*F*).

### Multiplicative Integration of Information about OMA and Visual Format.

**Fig. 3.**
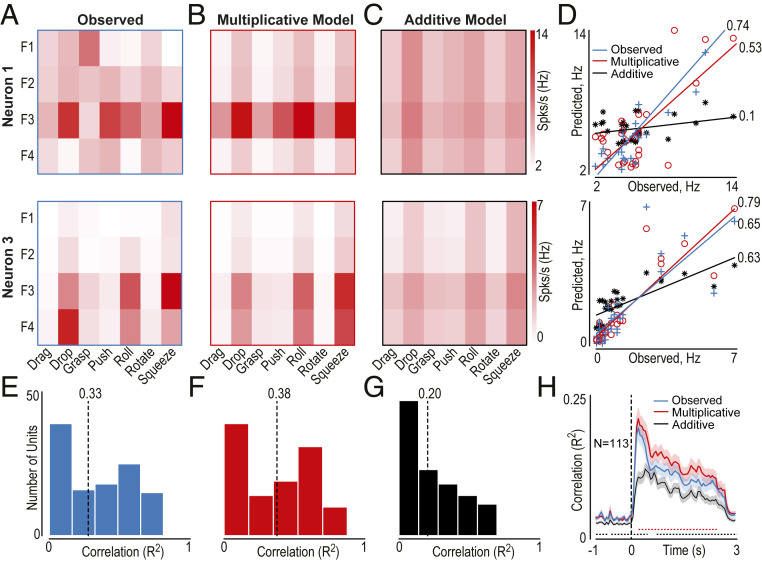
Multiplicative vs. additive mixing of OMA identity with visual format information. (*A*) Observed response matrix with half of the trials of two neurons (neurons 1 and 3) shown in Fig. 1*C*. F1 to F4 indicate the four formats (stand lateral, sit lateral, stand frontal, sit frontal), whereas the horizontal axis indicates the OMA exemplars. (*B* and *C*) Predicted responses for the multiplicative and additive models built on the other half of the trials of the same two neurons. (*D*) Observed response plotted against the response predicted with each of the models (multiplicative, additive) and the response observed with the other half of the trials (observed). Correlations between a split of the real data and observed (blue), multiplicative (red), and additive (black) model data. The values at the extremity of each line indicate *R*^2^. (*E*–*G*) Distribution of *R*^2^ between half of the trials and the other half of observed data (*E*), multiplicative model data (*F*), and additive model data (*G*). Dashed lines represent the median *R*^2^ value (indicated above). (*H*) Time-resolved correlation analysis (time bin, 150 ms; step, 50 ms) between a random split half of the real data and 1) the remaining data (blue), 2) multiplicative model data (red), and 3) additive model data (black) for the entire OMA-selective population. Colored dashed lines highlight the significant bins (only series of at least four consecutive bins with *P* < 0.05 are shown) for the comparison of each model with observed data.

Next, we asked how information about OMA identity and format in the main experiment integrates in the AIP at the individual-unit level. To answer this question, we adopted a model-fitting approach recently used to assess the combination of static information about object identity and image attributes in inferotemporal neurons (5). As an example, the responses of neurons 1 and 3 shown in Fig. 1*C* have been used to investigate how information about OMA and format integrates in the AIP. By fitting half of the trials of each unit (Fig. 3*A*) with a multiplicative (Fig. 3*B*) and an additive (Fig. 3*C*) model of their firing built on the basis of the other half of the trials (*Materials and Methods*), we found that the correlation between real data and the multiplicative model was highly significant and as good as the one between the two halves of the real data (Fig. 3*D*). Indeed, the correlation coefficients between the two halves of the observed data (Fig. 3*E*, median value 0.33) were slightly lower than those obtained between half of the observed data and the multiplicative model data (Fig. 3*F*, median value 0.38, Mann–Whitney *U* test, Z = 2.19, *P* = 0.028), indicating that the multiplicative model is a good predictor of single-unit tuning. In contrast, the additive model (Fig. 3*G*, median value 0.20) was a less accurate predictor than both observed data (Mann–Whitney *U* test, *Z* = 5.17, *P* = 3.13e^−7^) and the multiplicative model data (Mann–Whitney *U* test, *Z* = 6.81, *P* = 9.49e^−12^). Since OMAs are intrinsically dynamic stimuli which evolve over time, we also applied this procedure in a time-resolved manner (*Materials and Methods* and Fig. 3*H*), demonstrating that the multiplicative model was a steadily better predictor of AIP neural response during OMA unfolding than the additive model. Furthermore, the prediction of the multiplicative model was slightly better than that obtained with random split halves of the real data, as previously observed in inferotemporal neurons with static stimuli (5).

Altogether, these findings indicate that signals concerning identity and visual format are multiplicatively and dynamically combined in AIP neuronal activity during action observation.

### Decoding of Visual Format and OMA Identity from AIP Population Activity.

The multiplicative mixing of signals about visual format and OMA identity evidenced so far raises the issue of whether a stable readout of OMA identity is possible across formats. To address this issue, we trained a classifier to decode OMAs with the activity of all units recorded in one format and tested its decoding performance in the other formats (26). Consistent with single-neuron examples (Fig. 1*C*), this cross-decoding approach yielded poor classification accuracy (*SI Appendix*, Fig. S3), suggesting that the representation of OMA in the AIP is mainly format-specific.

**Fig. 4.**
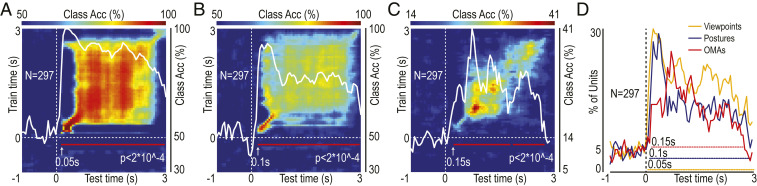
Population decoding of viewpoint, actor’s body posture, and OMA exemplar. (*A*) Classification accuracy of viewpoint (lateral and frontal, regardless of actor’s body posture and OMA). (*B*) Classification accuracy of actor’s body posture (standing and sitting, regardless of viewpoint and OMA). (*C*) Classification accuracy of OMA exemplars (regardless of the format). The decoding accuracy (color-coded) is expressed as a function of test and train time. The superimposed white lines represent the classification accuracy along the diagonal (scale; *Right*); the red lines indicate the period during which the decoding accuracy is significantly above chance level (beginning of the first 150-ms significant bin; *Materials and Methods*). (*D*) Percentage of units (out of the entire population) showing significant tuning for viewpoint, actor’s body posture, and OMAs (repeated-measures ANOVAs in 150-ms bins shifted forward in steps of 50 ms). Colored dashed lines highlight bins with a fraction of tuned units significantly higher than during baseline (χ^2^, *P* < 0.05).

Indeed, by training a classifier with the activity of all of the recorded units to discriminate among formats, both viewpoint (Fig. 4*A*) and posture (Fig. 4*B*) could be efficiently decoded irrespective of OMA exemplars. The square-shaped increase of their classification accuracy following stimulus onset indicates that the representation of viewpoint and posture is essentially static, coherent with the stationary nature of this visual information. The tuning for the viewpoint rose significantly above chance level from 50 ms after video onset (using a bin width of 150 ms), immediately followed by tuning for actor’s body posture at 100 ms after video onset. Then, we applied the same procedure to decode OMA identity by training and then testing a classifier in a format-independent decoding of OMA exemplars, obtaining a highly significant classification accuracy (Fig. 4*C*) at 150 ms after video onset. The population code showed in this case an essentially dynamic representation of OMAs, as demonstrated by the diagonal distribution of high decoding accuracy, which is consistent with the dynamic nature of this visual information. Importantly, the sequential coding of viewpoint, posture, and, finally, OMA identity in the AIP revealed with the neural decoding approach was confirmed, with the same timing, by comparing the fraction of units tuned to each factor relative to baseline during video unfolding (Fig. 4*D*).

### Readout of OMA Identity Depends on AIP Units with across-Format Encoding Stability.

A possible explanation for the superior performance of format-independent decoding of OMAs relative to cross-decoding (*SI Appendix*, Fig. S3) may be that individual units maintain a relatively stable ranking of OMAs across formats, despite rescaling in firing rate across formats (e.g., neurons 2 and 3 in Fig. 1*C*).

**Fig. 5.**
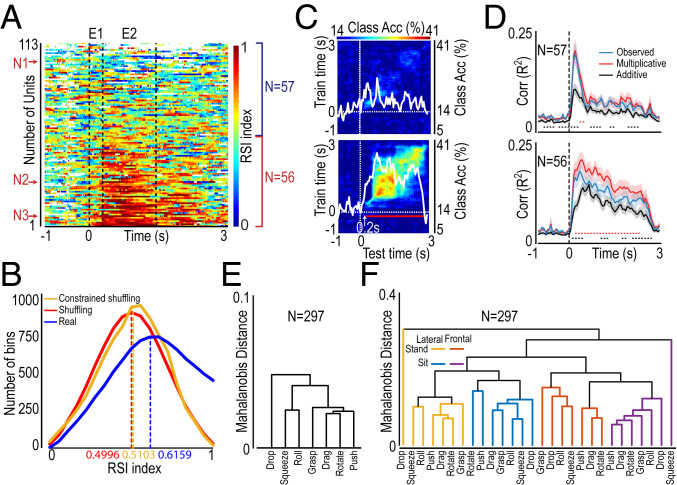
Stable and unstable OMA-selective units and clustering of OMA exemplars. (*A*) Time-resolved, across-format OMA rank stability index calculated for all OMA-selective neurons. For each bin, the color code represents the consistency across formats in the rank of the best OMA (rank 1) in the reference format (i.e., stand lateral). Units are arranged from *Bottom* to *Top* as a function of their total RSI score in epochs 1 and 2 (combined, greater on *Bottom*). We classified the first 56 units as stable and the last 57 as unstable. Labels of N1 to N3 (*Left*) indicate the position of the neurons (from 1 to 3) illustrated in Fig. 1*C*. (*B*) Frequency distribution of RSI values (in individual bins) obtained with real data, shuffling, and constrained shuffling of the data (*Materials and Methods*) during the interval corresponding to epochs 1 and 2, combined. Dashed lines represent the mean values of each distribution. (*C*) Format-independent decoding of OMA exemplars as a function of training and test time carried out on stable (*n* = 56) and unstable (*n* = 57) units defined based on RSI score (A). Conventions are as in Fig. 4. (*D*) Time-resolved correlation analysis between the multiplicative model (red), additive model (black), and observed data (blue) carried out on unstable (*Top*) and stable (*Bottom*) units, separately. Conventions are as in Fig. 3*H*. (*E*) Clustering of OMA exemplars based on the entire dataset, irrespective of the video presentation format. (*F*) Clustering of OMA exemplars based on the entire dataset, considering the video presentation format.

To test this hypothesis, we computed a time-resolved across-format OMA rank stability index (RSI; *Materials and Methods*), which is insensitive to firing-rate rescaling across formats, for each OMA-selective unit (Fig. 5*A*). The RSI ranges from 0 to 1, where a score of 1 is obtained when the best OMA exemplar in the reference format (stand lateral) is the preferred exemplar also in all of the other formats, and a score of 0 is obtained when the best OMA exemplar in the reference format is the less preferred (worst) in all of the other formats (the findings did not depend on the format used as reference; *SI Appendix*, Fig. S4*A*). The chance value of the RSI corresponds to approximately 0.5, obtained when the RSI is calculated on shuffled data (*Materials and Methods* and *SI Appendix*, Fig. S4*B*), and it is significantly different from RSI distribution calculated on real data (*t* = 36.2, *P* < 0.001). Importantly, the distribution of the RSI obtained from real data also differs from that obtained with a constrained shuffling of the data (*t* = 39.5, *P* < 0.001), which preserves the temporal structure of OMA selectivity changes in each test format while allowing us to test the possibility that the best OMA in the reference format remains the same across test formats only by chance (*Materials and Methods* and *SI Appendix*, Fig. S4*C*). The results clearly indicate that the across-format stability of OMA selectivity is significantly higher than that expected by chance (Fig. 5*B*). By ordering OMA-selective units based on their total RSI score calculated on epochs 1 and 2 (combined, highest values in Fig. 5*A*, *Bottom*), we classified the first 56 units as relatively stable and the last 57 units as relatively unstable. The single-neuron examples shown in Fig. 1*C* are indicated with red arrows in Fig. 5*A* (N1 to N3). It is clear that format-independent decoding of OMAs (Fig. 4*C*) critically depends on the contribution of stable units, whereas unstable units do not significantly add accuracy to the classification (Fig. 5*C*).

It can be argued that greater RSI values of stable units are an epiphenomenon of a greater presence of single units, but we found no significant difference in the prevalence of single units relative to multiunits between the stable and unstable sets (14 single-units for stable and 18 for unstable, χ^2^ = 0.14, *P* = 0.7). Alternatively, the greater RSI values of stable units could be simply a consequence of their overall greater OMA selectivity, but even this hypothesis is ruled out by the finding that OMA preference index values of the two sets of units are not significantly different during the video unfolding period (*SI Appendix*, Fig. S4*D*). Importantly, compared with unstable units, stable units also yielded more accurate decoding of both viewpoint and actor’s body posture (*SI Appendix*, Fig. S5), showing that this set of units conveys format-specific signals in addition to information on OMA exemplars. Indeed, by applying the above-described model-fitting procedure to stable and unstable units (separately) in a time-resolved manner (Fig. 5*D*), we found that in both sets of units the multiplicative model (red) predicted the data with higher accuracy than the additive model (black) and similar to randomly selected split halves of trials of the same units (blue). However, only stable units maintained this correlation pattern over the entire duration of the video.

These findings suggest that stable OMA-selective units multiplicatively integrate static signals about the visual format and dynamic signals about OMA identity along the whole action unfolding period, thereby making a specific contribution to the emergence of the above-described population code for OMAs across formats.

### Across-Format Representation of Individual OMA Exemplars in the Neural Space.

As a final step, we investigated the possible presence of a general and stable relationship between neural representations of specific OMA exemplars across formats. To do so, we performed a cluster analysis by computing the mean Mahalanobis linkage distance between exemplars using all of the available neural data in both a format-independent (Fig. 5*E*) and format-dependent manner (Fig. 5*F*). The results show that, independent of the format (Fig. 5*E*), grasp, rotate, drag, and push are typically closer to each other and segregate with respect to drop, roll, and squeeze. Interestingly, whereas actions belonging to the first cluster share the feature of being directed to an object initially lying on the table, actions of the second cluster share the initial presence of the object in the actor’s hand (although in dropping actions it is suddenly released and falls down, making this action the most distinguishable among all of the others). This relationship between OMA exemplars is generally maintained even within formats (Fig. 5*F*), although viewpoint appears to be the most clear-cut clustering factor (see the color code in Fig. 5*F*).

## Discussion

In this study, we investigated the neural mechanisms underlying a possible role of the AIP, an important hub in the observed-action network (11, 27), in the emergence of visual invariance, which is a crucial property of action perception. We found that the AIP neuronal population can robustly and independently encode both the visual presentation formats (viewpoint and posture) and OMA identity, but we did not find OMA-selective single neurons with fully invariant discharge across formats. Nonetheless, we revealed the existence of a set of units that maintain a stable OMA selectivity across-format, despite rescaling their firing rate in response to changes in the action’s visual format. Furthermore, we showed that AIP stable units can dynamically integrate information about the visual format and observed action in a multiplicative manner during video unfolding, allowing the emergence of a robust population code that represents OMA identity across formats.

In this study, we extended previous findings (11) by presenting monkeys with OMA exemplars performed by an actor in different visual formats, resulting from the combination of two different body postures and two viewpoints. Clearly, combining these two main factors yielded stimuli that differed for several additional visual features, such as the overall contrast of the stimuli or the presence/absence of the agent’s whole body. In fact, the goal of the study was not to precisely isolate the effects of these factors but rather to test the possible presence and robustness of an invariant representation of OMAs in the AIP across formats. We found that despite the visual differences among the four formats, the fraction of OMA-selective vs. unselective and unresponsive units did not vary among formats; furthermore, OMA-selective units’ response showed negligible correlations with the amount of motion in the videos. Taken together, these findings suggest that low-level visual features appear to have little influence on OMA selectivity.

Nonetheless, to test directly the tuning of AIP neurons for OMAs rather than visual features, we performed a control experiment in which we presented the monkey with a variety of videos portraying natural dynamic stimuli, including manual actions (groom and grasp). We found that none of the recorded units was influenced by the amount of contrast in the stimuli, whereas 15% of them showed an overall tuning for the amount of motion in the videos, which is in line with the generally weak but systematically observed connections of the AIP with areas of the dorsal stream, such as the medial superior temporal area (11, 28, 29); indeed, this latter area is known to play a crucial role in the processing of visual motion (30, 31). By removing this small set of units from further analysis, we could provide conclusive evidence that a majority of AIP units exhibiting selectivity for the videos showed a preferential tuning for manual actions relative to the other tested stimuli, in line with previous indirect evidence in humans (23, 24). This constitutes a direct demonstration that the AIP has a preference for OMAs among many other types of natural dynamic stimuli, which so far have not been used in any neurophysiological study of this area (18, 32).

Remarkably, we found no evidence of fully invariant representation of OMA across formats, neither at the individual-unit nor at the population level (1–3). Instead, we found that AIP neurons combine information multiplicatively on OMA identity and visual format, thereby allowing us to separately decode each type of signal independent from the other. Similar computational principles, in which high- and low-level visual features are encoded by the same neuronal population, have been previously shown to be at the basis of static object and image processing in the inferotemporal cortex (5, 33, 34). Indeed, in the present study, we found that the visual format could be accurately decoded from the whole AIP population activity by training and testing a classifier to decode viewpoint, which was significant at 50 ms after video onset (in bins of 150 ms), and actor’s body posture, which could be decoded with significant accuracy shortly after, at 100 ms from video onset. In addition, we found significant classification accuracy of OMAs in a format-independent decoding, at 150 ms after video onset, demonstrating that despite a variety of additional visual features characterizing the formats, a signal concerning OMA identity can be extracted from AIP neuronal activity. How may format-independent representation of OMAs be achieved in the AIP?

To address this issue, we quantitatively evaluated the extent to which the preference of OMA-selective units during video presentation remains the same or changes across formats. We identified a set of AIP units with a temporally stable, across-format preference for OMAs. These properties cannot be accounted for by spurious factors, such as differences in the proportion of single units to multiunits or the degree of preference for OMA exemplars between stable and unstable units, allowing us to conclude that stable units do in fact contribute to a format-invariant OMA identity signal. In the attempt to better understand how they can generate this signal, we found that stable units revealed stronger and much more sustained correlation with a multiplicative model of their discharge during video unfolding relative to unstable units. It is noteworthy that the multiplicative model also predicts real data better than the additive one, even for unstable units, which therefore mix OMA and format information as well but without comparable across-format stability. Indeed, stable units provide higher decoding accuracy not only for OMA identity but also for viewpoint and actor’s body posture relative to unstable units. Thus, these findings indicate that multiplicative mixing of static signals about the visual format and of dynamic signals about OMA identity lead to the emergence of a population code for OMA identity that is stable across formats despite the considerable rescaling of neuronal firing rate.

Although speculative, we propose that the representation of specific OMA exemplars in the AIP may derive, to some extent, from the extraction of functional relationships between the biological effector (the actor’s hand) and the target object. Indeed, by measuring the distances between individual OMA exemplars in the neural space, it appears that the actions in which the hand moves toward a target lying on a table segregate from the actions in which the hand is already in contact with the target before their onset, both across and within visual formats. Previous studies of the visuomotor properties of AIP neurons showed that, during planning and execution of grasping actions, a fundamental coding principle of the AIP consists of the extraction of the dynamic relationship between one’s own hand and the target (18, 32, 35–40). Here we show that a similar coding principle can also apply to the visual processing of manipulative actions of others, thereby providing a way to assess the result of observed actions.

Our study suggests that by multiplicatively mixing the dynamic signals about the specificities of individual OMAs with static information about their visual format, AIP neurons preserve a stable relationship between OMA exemplars despite the huge variability in their peripheral visual features, thereby allowing a stable readout of observed-action identity.

## Materials and Methods

### Experimental Model and Subject Details.

Experiments were carried out on two purpose-bred, socially housed adult *Macaca mulatta*, one female (Mk1, 4 kg) and one male (Mk2, 7 kg). Before recordings, the monkeys were habituated to sit in a primate chair and to interact with the experimenters. Using positive reinforcement techniques, they were then trained to perform the tasks described in a previous study (11) as well as the visual fixation task employed for the present study. When the training was completed, a head fixation system was implanted under general anesthesia (ketamine hydrochloride, 5 mg/kg intramuscularly [i.m.] and medetomidine hydrochloride, 0.1 mg/kg i.m.), followed by postsurgical pain medications. Surgical procedures were the same as previously described in detail (41). All experimental protocols complied with the European law on the humane care and use of laboratory animals (Directives 86/609/EEC, 2003/65/CE, and 2010/63/EU), were approved by the Veterinarian Animal Care and Use Committee of the University of Parma (Protocol 78/12 17/07/2012), and were authorized by the Italian Ministry of Health (Decreto Ministeriale 294/2012-C, 11/12/2012).

### Apparatus and Behavioral Paradigm.

During the video presentation, the monkey was seated on a primate chair in front of a monitor (1,920 × 1,080 pixels, 60 Hz) located 57 cm from the monkey’s face. The video took up an area of 13.04° × 9.85° of the visual field in the horizontal and vertical dimensions, respectively. The observation tasks were automatically controlled and monitored by LabView-based software, enabling the interruption of the trial if the monkey broke fixation, released the starting button, or did not respect the task temporal constraints. In all these cases, no reward was delivered. After correct completion of a trial, the monkey was automatically rewarded with the same amount of juice in all conditions (pressure reward delivery system; Crist Instruments).

#### OMA videos: Main experiment.

The videos used in the main study portrayed seven different OMA exemplars (drag, drop, grasp, push, roll, rotate, squeeze). Each OMA exemplar was presented in four visual formats, resulting from the combination of two actor’s body postures (standing, sitting) and two viewpoints (lateral, frontal). The videos from the lateral and frontal viewpoints were acquired simultaneously by using two identical cameras operating in parallel (42); thus, they portrayed the very same action filmed from different perspectives. As a consequence, different parts of the body were visible, as would be the case for static stimuli, and different directions of the actions in space were aligned with the visual axis. The viewpoint change also implied changes in the background against which the action appeared (the wall of the room vs. the chest of the agent). The videos of different actor’s body postures were taken from the same camera positions, and the actors performed the actions as similarly as possible while recoding OMAs in standing and sitting positions. As camera position did not change, the camera angle and the distance from the camera to the action were slightly different, inducing some deformation and reduction of the images in the sitting condition. In this setting, each OMA was performed by two actors (one male and one female), on two target objects (of the same size and different color), providing four different variants of each of the seven OMA exemplars in each of the four formats, for a total of 112 distinct videos. An example of the OMA exemplars administered in the four visual formats is shown in Fig. 1*A*. Each individual video was presented three times, for a total of 336 videos presented in each session. Hence, each individual combination of OMA and format was presented 12 times, considering the gender of the actor and the two target objects as additional sources of variability in the visual features of the videos, which makes more robust the possible differences observed in the coding of OMA exemplars. The sequence of task events required the monkey to gaze at a red square on a scrambled background. Then, the video stimulus started and lasted 2.6 s. The monkey was required only to remain still, with its hand on the starting button, and to maintain fixation for the entire duration of the trial. If the monkey maintained fixation within a 3° spatial window centered on the fixation point for the entire duration of the trial, a reward (a drop of fruit juice) was automatically delivered.

#### Natural video stimuli: Control experiment.

The videos used in the additional control experiment portrayed nine different natural dynamic scenes, including monkey manipulative actions (grasp and groom), emotional facial gestures (lip smack and scream), neutral facial gestures (yawn and chew), monkey at rest, moving animal, and dynamic landscape. Examples of frames taken from the crucial dynamic part of each video are shown in Fig. 2*A*. Two of the videos did not portray any monkey. The moving animal was a crab rapidly walking at the center of the scene and followed by the camera, whereas the landscape video showed a panning shot of a forest. All of the remaining stimuli portrayed rhesus macaques filmed at a field station either at rest or committed to perform different types of manual or facial actions. Because they were essentially natural and uncontrolled stimuli, videos varied considerably in the degree of contrast and amount of motion, allowing us to better study the possible effects of these low-level features on the neuronal discharge. The stimuli were presented on the same monitor and with the same procedure described above for OMA stimuli, but the monkey was not required to rigidly maintain fixation; in this case, it had to remain still and looking at the stimulus during the full length of the video (3.5 s). Nonetheless, being overtrained, the animal maintained fixation at the center of the screen for the entire duration of all videos, as revealed by the analysis of the recording of eye position (*SI Appendix*, Fig. S2*C*), although this was not explicitly required. Each of the nine stimuli was randomly presented for a total of 10 trials.

### Recording Techniques.

Neuronal recordings were performed by means of chronically implanted arrays of linear silicon probes with 32 recording channels per shaft. Probes were implanted by estimating the angle of penetration with MRI-based reconstruction of the outline of the intraparietal sulcus at the selected site of insertion (11). Previous reports provide more details on the methodology of probe fabrication, assembly, and implantation (43–45), as well as on probes’ recording performance over time in chronic applications (14).

The signal from the 128 channels was simultaneously amplified and sampled at 30 kHz with four 32-channel amplifier boards (Intan Technologies), controlled in parallel via the electrophysiology platform Open Ephys (http://open-ephys.org/). All formal signal analyses were performed offline with fully automated software, MountainSort (46), using −3.0 SDs of the signal-to-noise ratio of each channel as the threshold for detecting units. To distinguish single units from multiunits, we used the noise overlap, a parameter that can vary between 0 and 1, with units with a value below 0.15 being considered as single. Single-unit isolation was further verified using standard criteria (interspike interval distribution, refractory period > 1 ms, and absence of cross-correlated firing with a time lag of ∼0 relative to other isolated units, to avoid oversampling), possible artifacts were removed (with 3 SDs from the averaged sorted waveform), and all of the remaining waveforms that could not be classified as single units formed the multiunit activity.

### Recording of Behavioral Events and Definition of Epochs of Interest.

A contact-sensitive device (Crist Instruments) was used to detect when the monkey’s hand (grounded) touched the metal surface of the starting button or detached from it. The signal was used by LabView-based software to monitor the monkey’s behavior. The same software also controlled the presentation of the videos and eye position, monitored in parallel with neuronal activity with an eye-tracking system consisting of a 50-Hz charge-coupled device video camera equipped with an infrared filter and two spots of infrared light. An analog signal related to horizontal and vertical eye position was fed into a computer equipped with dedicated software, enabling calibration and basic processing of eye position signals. Signals related to eye movement and task events were recorded and stored together with the neuronal activity and subsequently used to construct the data files for statistical analysis.

For the analysis of the main experiment with OMAs, we considered the following epochs of interest: 1) baseline, 500 ms before video presentation onset; 2) epoch 1, 300 ms from video onset; and 3) epoch 2, including the subsequent 1,200 ms of the video. The choice of these two epochs was motivated by the fact that, according to a previous study (11), epoch 1 included primarily static information about the depicted action conveyed by the actor’s initial body posture, whereas epoch 2 provides all of the dynamic information required to unambiguously identify the observed action (see also *SI Appendix*, Fig. S1*E*). The duration of epoch 2 balances in the best possible way the unavoidably different duration of OMAs.

For the analysis of the control experiment with natural video stimuli, we considered the following epochs of interest: 1) baseline, 500 ms before video presentation onset; and 2) video, from 500 to 3,000 ms after the video onset. Removing the first 500 ms from the analyzed time window has been necessary because in this putatively free-gaze experiment the analysis of eye position during video presentation (*SI Appendix*, Fig. S2*C*) revealed that the monkey gazed at the center of the screen within the first 500 ms. Thus, excluding this period ensures that eye movement has a negligible role in influencing neuronal response.

### Quantification and Statistical Analysis.

All analyses were carried out using MATLAB 2018b and Statistica v.13 (StatSoft).

#### Classification of units.

In the main experiment, units (single and multi) were classified primarily on the basis of possible modulation of their activity in epochs 1 and/or 2 of video presentation relative to baseline, according to our previous study (11). The analysis was performed by means of a 3 × 7 repeated-measures ANOVA (factors: epoch and exemplar) carried out separately in each format and followed by Bonferroni post hoc tests, where appropriate. We classified as action-related all units showing a significant effect (*P* < 0.05) of the factor epoch (epochs 1 and/or 2 relative to baseline), either as a main or interaction effect with the factor exemplar, in at least one of the four visual formats. The remaining units were classified as task-unrelated. Action-related units showing, in addition, a significant effect (*P* < 0.05) of the factor exemplar, either as a main or interaction effect with the factor epoch, were classified as OMA-selective.

In the control experiment, units (single and multi) were classified on the basis of possible modulation of their activity in the video epoch relative to baseline. The analysis was performed by means of a 2 × 9 repeated-measures ANOVA (factors: epoch and video) followed by a Fisher LSD test. All units showing a significant effect (*P* < 0.05) of the factor epoch, either as a main or interaction effect with the factor video, were classified as video-related. Then, video-related units showing, in addition, a significant effect of the factor video, either as a main or interaction effect with the factor epoch, were considered selective.

All final population plots were made using a bin size of 60 ms and steps of 20 ms. The time course of population net activity is obtained by subtracting the mean activity value calculated during baseline from each bin along the trial of each neuron to be included in the population plot.

#### Decoding analyses.

The methodology employed for the decoding analysis was that described by Meyers (26) and used in our previous studies (11, 16). Specifically, we assessed the decoding accuracy of a Poisson naïve Bayes classifier trained and tested to classify a single factor, such as OMA or format, regardless of the other (Figs. 4 and 5*C*). We also used cross-decoding to test whether and to what extent the classifier trained to decode OMAs in one format can generalize its performance to decode OMAs from the data collected in another format (*SI Appendix*, Fig. S3; cross-decoding).

Regardless of the decoded factor, for each neuron, data were first converted from raster format into binned format. Specifically, we created binned data that contained the average firing rate in 150-ms bins sampled at 50-ms intervals for each trial (data point). We obtained a population of binned data characterized by a number of data points corresponding to the number of trials × conditions (i.e., 48 × 7 = 336 data points for format-independent OMA decoding; 24 × 7 = 168 data points for viewpoint and posture decoding; 12 × 7 = 84 data points for OMA cross-decoding) in an *N*-dimensional space (where *N* is the total number of neurons considered for each analysis). Next, we randomly grouped all of the available data points into a number of splits corresponding to the number of data points per condition, with each split containing a “pseudopopulation,” that is, a population of neurons that were partially recorded separately but treated as if they were recorded simultaneously. Before sending the data to the classifier, they were normalized by means of *z*-score conversion so that neurons with higher levels of activity did not dominate the decoding procedure. Subsequently, the classifier was trained using all but one of the splits of the data and then tested on the remaining one. This procedure was repeated as many times as the number of splits (i.e., 48 in the case of format-independent OMA decoding; 24 in the case of format—viewpoint or posture—decoding; 12 in the case of OMA cross-decoding), leaving out a different test split each time. To increase the robustness of the results, the overall decoding procedure was run 50 times with different data in the training and test splits, and the decoding accuracy from all these runs was then averaged. All of the analyses were performed on data collected from the two monkeys.

Note that when this procedure is applied by training the classifier to decode a specific isolated factor (i.e., OMA in one format) and testing its decoding performance in another condition (i.e., one remaining format), the results of this cross-decoding provide information on the generalization of the population code (*SI Appendix*, Fig. S3).

To assess whether the classification accuracy in the various analyses was above chance, we ran a permutation test using the Neural Decoding Toolbox (26) in which the decoding analysis was run with the labels to be classified randomly shuffled, in order to obtain a null distribution to be compared with the accuracy of the decoding carried out on real data. The shuffling/decoding procedure was run 50 times. The *P* value was found by assessing, at each point in time, how many of the points in the null distribution were greater than those in the real decoding distribution and selecting only periods of at least four consecutive significant bins to visualize significant decoding accuracy in the plots (Figs. 4 and 5 and *SI Appendix*, Figs. S3 and S5). The decoding results were considered statistically significant only if they were greater than all of the shuffled data tested in the null distribution (decoding accuracy was plotted with 150-ms bins shifted forward in steps of 50 ms). Each data point is plotted in correspondence with the beginning of the reference interval of that bin (i.e., time 0 refers to data in a bin ranging from 0 to 150 ms).

#### Model fitting.

To compare the way in which AIP units combine information on visual format and OMA identity, we first created neural response matrix *O* (Fig. 3*A*) taking the firing rate of each unit during epoch 2 of video presentation in even-numbered trials, with entries *O*_*ij*_ representing the response to the *i*^th^ OMA exemplar in the *j*^th^ visual format. Since we have seven OMA exemplars and four formats, the response matrix *O* has seven columns and four rows. Then, we fit this response matrix to two models (5) and contrasted the results with a cross-validation procedure between even- and odd-numbered trials, as reported below:

1) Additive model. To compute the additive model for any given unit, we started from the neural response matrix *O* and computed the two constants *k*_1_ and *k*_2_ by applying linear regression. Then, to generate the new matrix based on the additive model (*A*; Fig. 3*C*), we averaged the activation response matrix *O′* constituted by odd-numbered trials along the rows to obtain the format activation [*F*_1_ to *F*_*j*_] and along the columns to obtain the OMA exemplar activation [*E*_1_ to *E*_*i*_]. Then, the additive model prediction can be written as *A*_*ij*_ = *k*_1_*E*_*i*_ + *k*_2_*F*_*j*_, where *k*_1_ and *k*_2_ represent the constants defined above, *E*_*i*_ represents the average response for each OMA exemplar (in columns) regardless of the format, and *F*_*j*_ represents the average response for each format (in rows) regardless of the exemplar.
2) Multiplicative model. The response matrix of the multiplicative model (*M*; Fig. 3*B*) can be obtained as a product of the same two factors (*M*_*ij*_ = *E*_i*_F_*j*_) estimated by using singular value decomposition (SVD) according to previous studies (5, 47). Thus, we applied SVD to the response matrix *O′* (constituted by odd-numbered trials) as *O′* = *U*Σ*V*, where *U* and *V* are matrices containing the left and right singular vectors, respectively, and Σ is a diagonal matrix containing the singular values. Next, we calculated the multiplicative model prediction (Fig. 3*B*) for each *i*^th^ neuron as *M*_*i*_ = *u*_*i*_*s*_*i*_*v*_*i*_^*T*^, where *u*_*i*_ and the transposition of *v*_*i*_ are the first column vectors of the *U* and *V* matrices, respectively, and *s*_*i*_ is the first entry of the diagonal matrix Σ.

To quantify the accuracy in the models’ prediction of each neuron response pattern, we linearly correlated its matrix *O* with the *A* (additive), *M* (multiplicative), and *O′* (observed) matrices, using the result of this latter procedure as an index of similarity between split halves of the actual data. Each resulting *R*^2^ value (Fig. 3*D*) quantifies the similarity between observed values and those predicted by each of the models.

Since OMAs are intrinsically dynamic stimuli, which evolve over time, we also applied this procedure in a time-resolved manner (Figs. 3*H* and 5*D*) by calculating additive and multiplicative models with a random split half of the data included in 150-ms windows shifted in steps of 50 ms. For each step, we calculated the correlation value (*R*^2^) between the models and the response matrix *O* or between the two halves of the observed data and ran this procedure 20 times, using each time different random selections of the data to generate and then validate the model. The plots (Figs. 3*H* and 5*D*) report the average *R*^2^ value obtained for each unit at each time point, and differences between the curves have been tested with a sliding Wilcoxon test (*P* < 0.05).

#### Rank stability index.

The RSI was calculated for each of the 113 OMA-selective units (averaged activity across trials) by first identifying, in a reference format (Fig. 5*A* and *SI Appendix*, Fig. S4), the preferred exemplar (rank 1) over the seven tested OMAs in 500-ms bins, shifted forward in steps of 20 ms along the entire task unfolding period. Then, we assessed the raw RSI value (*RSIr*) in the corresponding bin of each test format (*i* = 3), based on the following equation:

$$RSIr=1+\sum_{i=1}^{i=n}\left(1-\left(\frac{1}{7}*\left(Rf_{i}-1\right)\right)\right).$$

In this equation, the first constant value 1 represents the score assigned to the reference format (best OMA, rank 1); the second constant value 1 represents the maximum score obtainable in each test format (i.e., when the best OMA exemplar is the same as in the reference format); 1/7 represents the probability of finding as the preferred exemplar in the test format the same OMA ranked 1 in the reference format; *Rf*_*i*_ represents the observed rank (from 1 to 7) in the test format of the OMA ranked 1 in the reference format; the last constant value 1 corresponds to *Rf*_*i*_ = 1. *RSIr* values range between a minimum of 1.4287, where the best OMA exemplar in the reference format is the worst in all of the test formats, and a maximum of 4, where the best OMA exemplar in the reference format is the best in all test formats as well. The minimum *RSIr* value was then subtracted from each observed *RSIr* and the result was normalized relative to the maximum value, producing RSI values ranging from 0 to 1 with 19 intervals (Fig. 5*A* and *SI Appendix*, Fig. S4). The same procedure has also been applied by shuffling the *Rf*_*i*_ values of all bins in each format (Fig. 5*B* and *SI Appendix*, Fig. S4*B*), independently. Specifically, we substituted every *Rf*_*i*_ in each test format with a random integer value ranging from 1 to 7, generating totally random values (chance distribution). We also applied this procedure with an additional constraint, that is, by substituting a given *Rf*_*i*_ value (i.e., 1), wherever it occurred in all bins of a test format, with the same random integer value, ranging from 1 to 7. This constrained shuffling procedure (Fig. 5*B* and *SI Appendix*, Fig. S4*C*) allowed us to preserve the temporal structure of OMA selectivity changes while testing the possibility that the best OMA in each bin of the reference format remained the same across test formats by chance.

#### Hierarchical cluster analysis.

To find evidence for the possible relation between neural representations of OMA exemplars in the four visual formats, we performed a hierarchical cluster analysis. Given a population of *N* units, the firing rates of all units were calculated by binning the spiking activity and averaging it across trials. We created a firing-rate matrix *F* with *N* rows and *c*∙*t* columns (where *c* is the number of conditions and *t* is the number of time points per condition within the epoch of interest). Then, we computed the Mahalanobis linkage distances (MATLAB function: manova1) between the activities in the *N*-dimensional state space of all possible pairs of conditions in the epoch of interest. Because the Mahalanobis distance between any pair of arbitrarily selected conditions increases linearly as a function of the number of units in the population, the resulting matrix of distances was normalized by dividing it by *N*. Finally, the normalized distance matrix was used to create a hierarchical cluster tree based on the average linkage criterion (MATLAB function: manovacluster) and presenting the cluster solutions in the form of dendrograms. While building the dendrograms, we sorted the leafs within a branch based on their average distance to the nearest branches (MATLAB function: optimalleaforder).

### Data and Code Availability.

All data discussed in the paper are available in the main text and *SI Appendix*. We used standard MATLAB functions and publicly available software indicated in the manuscript for analysis.

## Supplementary Material

Supplementary File
