# Supplementary material for: Stable readout of observed actions from format-dependent activity of monkey’s anterior intraparietal neurons
Source: Proc Natl Acad Sci U S A. 2020 Jun 24;117(28):16596–605. doi: 10.1073/pnas.2007018117 (PMC7369316; doi:10.1073/pnas.2007018117)
Supplement: Supplementary File [file pnas.2007018117.sapp.pdf]

PNAS
www.pnas.org

Supporting Information for

Stable readout of observed actions from format-dependent activity of monkey's anterior intraparietal neurons

Marco Lanzilotto[a,b,1], Monica Maranesi[b], Alessandro Livi[b,c], Carolina Giulia Ferroni[b], Guy A. Orban[b,2], Luca Bonini[b,2,1]

[a] Department of Psychology, University of Turin, Via Verdi 10, 10124, Torino - Italy.

[b] Department of Medicine and Surgery, University of Parma, via Volturno 39, 43125, Parma - Italy.

[c] Department of Neuroscience, Washington University in St. Louis, St. Louis, MO, USA, 63110.

[2] G.A.O. and L.B. contributed equally to this work.

[1] To whom correspondence may be addressed. Email: marco.lanzilotto@unito.it or luca.bonini@unipr.it

www.pnas.org/cgi/doi/10.1073/pnas.2007018117

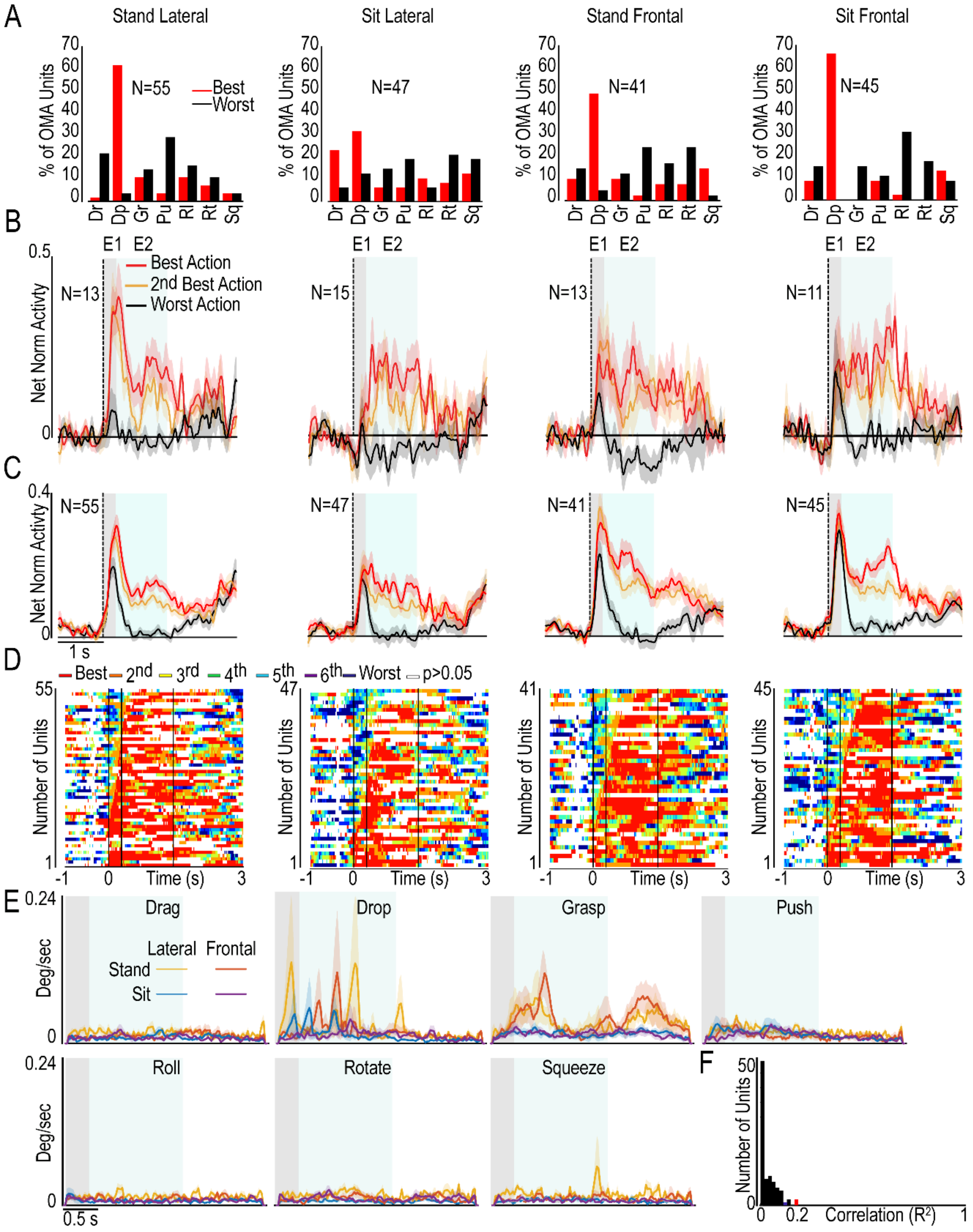

Fig. S1. (A) Percentage of units having each exemplar as best (red) or worst (black) OMA in each format (calculated in Epoch 2). (B) Time course of the net-normalized activity of OMA selective single-units. A 2x2x3 ANOVA (factors: Viewpoint, Posture, Epoch) carried out on the response of the best action (red curve) yielded significant main effect only for the factor Epoch (i.e. baseline vs activity during Epoch 1 and 2 averaged, $p < 0.001$, Bonferroni corrected). (C) Time course of the net-normalized activity of OMA-selective units (single- and multi-units together). Note that in each

format the plot includes a minority of units with suppressed discharge (7% in stand lateral, 15% in stand frontal, 17% in sit lateral, 11% in sit frontal). The same ANOVA applied in B yielded significant main effects for the factors Viewpoint (frontal stronger than lateral, $p = 0.007$) and Epoch (Epoch 1 and 2 stronger than baseline, $p < 0.001$), but not Posture. (D) Time resolved cross-validation of the best OMA exemplar (rank = 1) obtained with each unit's activity in Epoch 2 throughout the task-unfolding period (bin width 300ms, step 20ms). The color code represents, bin by bin, the local rank of the OMA ranking 1 in Epoch 2. Bins with neural activity that is not significantly different from baseline (sliding window ANOVA, bin width = 300ms, step = 20ms, $p > 0.05$ uncorrected) have been blanked out. E1, Epoch 1; E2, Epoch 2. Note that the preference for the specific OMA exemplar classified as best in Epoch 2 remained constant throughout the duration of the video presentation period in spite of the considerable differences in the magnitude of body-shape changes among the videos (shown in E). (E) Average speed (degree/second) for each OMA exemplar during the video presentation period in each of the four formats (color code), calculated by computing the speed variation (or optic flow) between successive frames of each individual video, according to the methodology described in a previous study (48). Each trace in the plot represents the average of the 4 variants of each video. (F) Distribution of R^2 values for the correlation between amount of motion (peak value for each video in Epoch 1 and 2) and peak of activity (maximum firing rate for each facilitated OMA selective unit during the same period). Note that the percentage of correlated unit is lower than 5% ($n = 2$, with $p = 0.02$ for both units).

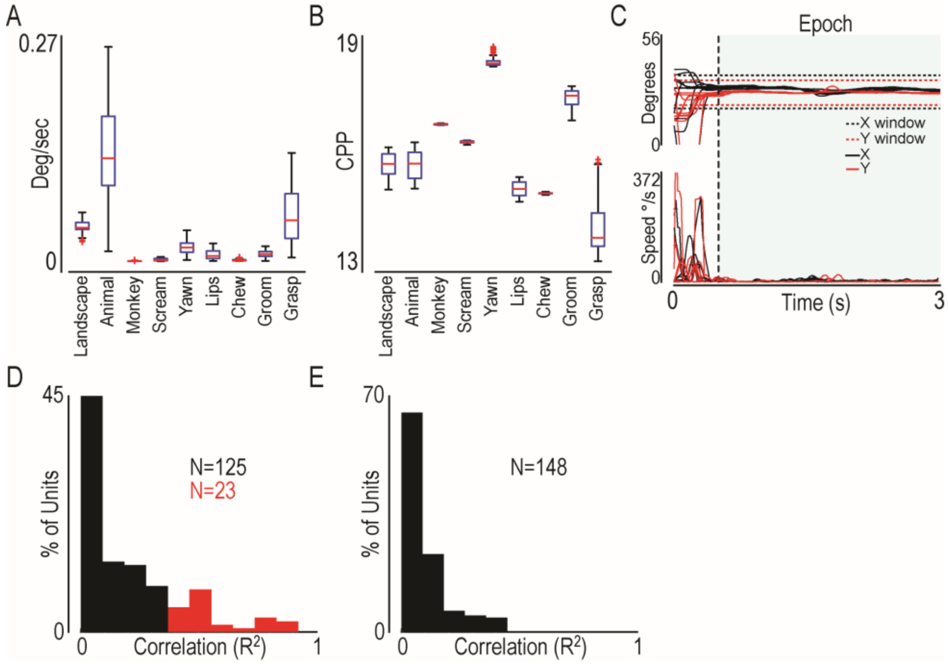

Fig. S2. (A) Median values for the amount of motion (deg/sec) in each video (horizontal red lines). The speed in each frame was averaged to produce the amount of motion for each video. The two ends of the rectangles indicate the 25th (lower) and 75th (upper) percentile of each distribution, and the bars show the absolute minimum and maximum values. It is clear that both in terms of magnitude and variability, grasp and groom are extremely different from each other and do not constitute outliers with respect to the other videos. (B) Median values of amount of contrast (Contrast Per Pixel, CPP) of each video (horizontal red lines). The CPP is computed by applying a sliding filter (Matlab function: conv2) to calculate the average luminance contrast between each pixel and the 8 adjacent pixels surrounding it. We run this procedure on every pixel of each frame, obtaining a single CPP value for each frame of a video by averaging the calculation results over all the pixels of a frame (49). (C) Representative recordings of the horizontal (X) and vertical (Y) eye position of Mk1 during the control experiment. Time 0 indicates the presentation onset of the video. The vertical dashed line corresponds to 500ms. The horizontal black and red lines correspond respectively to the horizontal and vertical external borders of the presented videos. Thus, the monkey brings its gaze in the area of the video presentation (16° x 11°) within 500ms and keep it fixed for the entire duration of the trial, as shown by ocular speed profile (bottom). (D) Correlation between the average amount of motion during the video presentation epoch shown in C and neuronal discharge during the same period. The distribution of the R^2 values is presented for all 148 neurons, of which 23 showed significant correlation and were not considered among video-selective neurons. (E) Correlation between the average amount of contrast (CPP) in the same epoch of the videos and neuronal discharge during the same period. No unit showed significant correlation between contrast and firing rate.

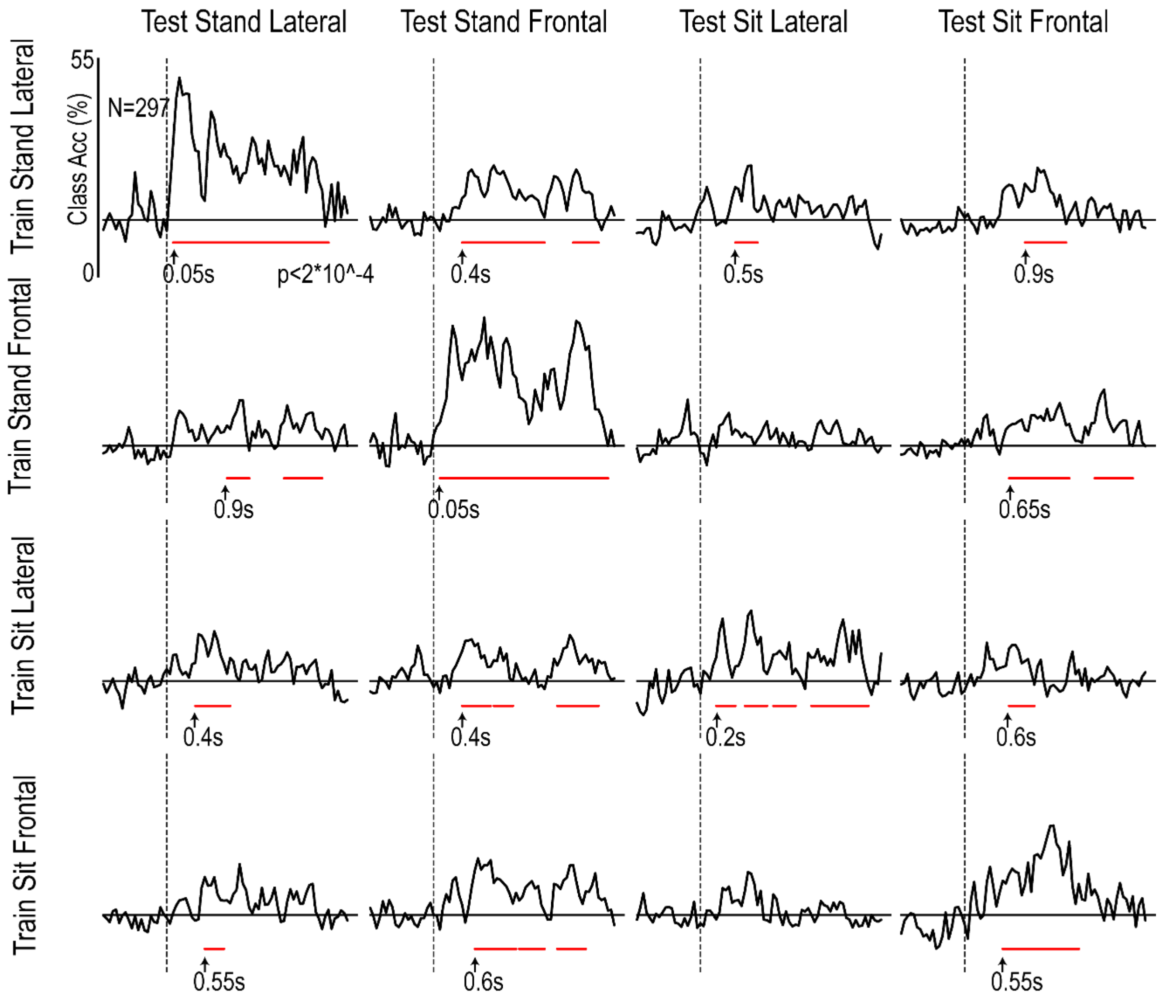

Fig. S3. Cross-decoding of OMAs during video presentation in each format. The diagonal (from top left to bottom right of the figure) represent the decoding accuracy when using the same set of data for training and testing. Red lines below each plot indicate the time period in which the decoding accuracy was significantly above chance. Arrows indicate the latency of significant classification accuracy onset relative to the beginning of the videos in that format.

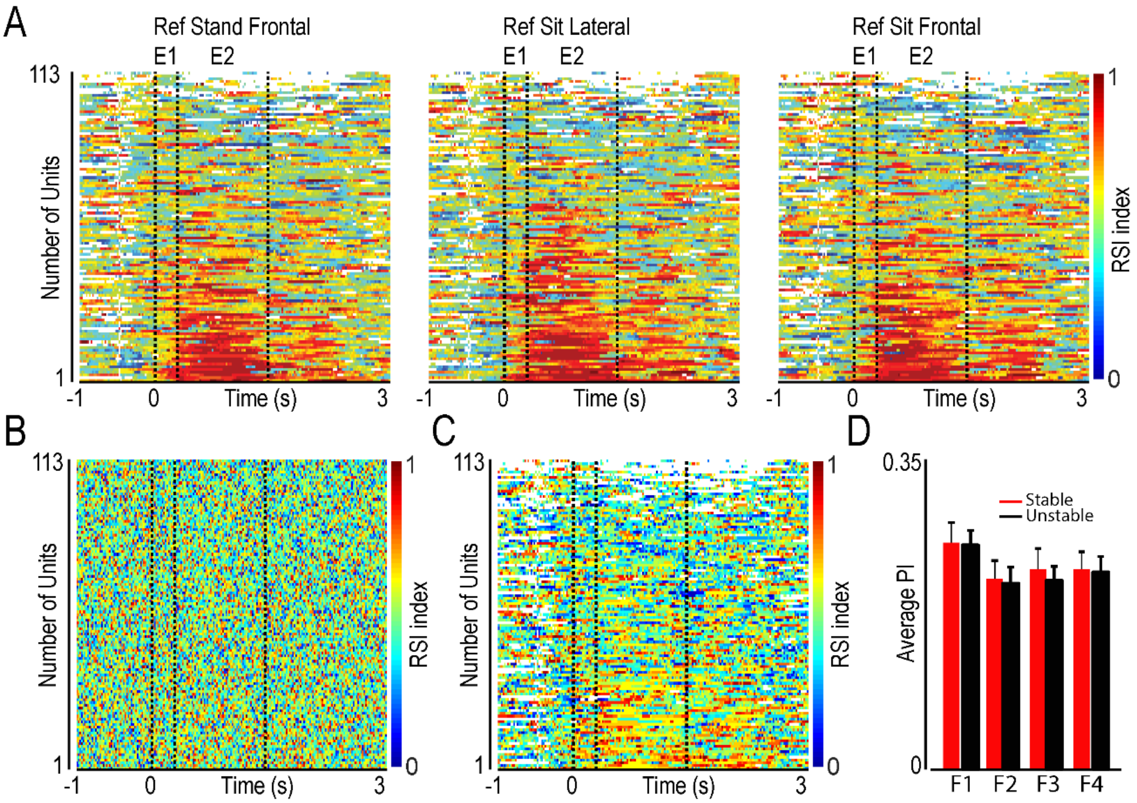

Fig. S4. (A) Time-resolved, across formats OMA rank stability index (RSI) calculated for all OMA-selective neurons by using each remaining format (besides the one shown in Fig. 5A) as reference format. Conventions as in Fig. 5. (B) Matrix of RSI data obtained by shuffling *Rfi* of OMA exemplars in each format. (C) Matrix of RSI data obtained by constrained shuffling of Rf_i in the test relative to the reference format, preserving temporal changes of selectivity but reducing the across-format stability to chance (see Methods). (D) Averaged PI calculated for each format (F1, stand lateral; F2, stand frontal; F3, sit lateral; F4, sit frontal) in stable (red) and unstable (black) class of units in the period encompassing Epoch 1 and 2. A 2x2x2 factorial ANOVA (factors: Class, Viewpoint, Posture) yielded no significant main or interaction effects. To compute OMA preference index (PI) we used the mean activity across trials in epoch 1 and 2 as input for the following equation:

$$PI_{OMA} = \frac{n - (\sum r_i / r_{pref})}{n - 1}$$

where n is the number of OMAs (n = 7), r_i is the unit response associated with OMA *i,* and r_{pref} is the unit response associated with the preferred OMA. The PI ranges from 0 to 1, with a value of 0 corresponding to identical response magnitude for all conditions and a value of 1 corresponding to a response to only one condition.

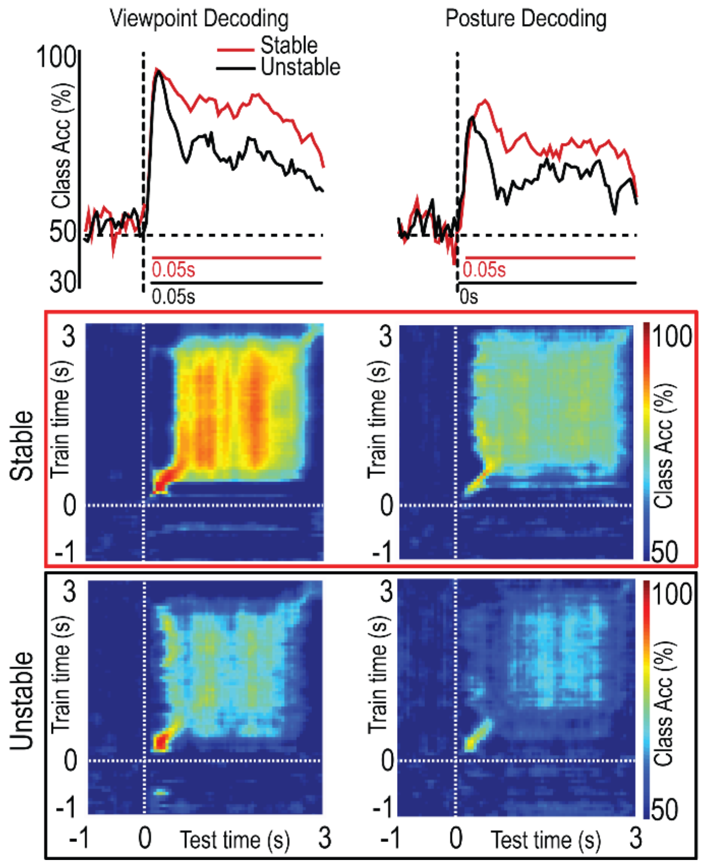

Fig. S5. Classification accuracy of agent's viewpoints and actor's body postures as a function of test and training time carried out on stable (N = 56) and unstable (N = 57) units defined based on RSI score (Fig. 5C). Conventions as in Fig. 4.

References

48. K. Pauwels, M. Van Hulle, Optic flow from unstable sequences through local velocity constancy maximization. *Image and Vision Computing* **27**, 579-587 (2009).

49. M. Eramian, D. Mould, "Histogram equalization using neighborhood metrics" in *The Second Canadian Conference on Computer and Robot Vision (CRV'05)*, (IEEE, 2005), pp. 397-404.
